# Elucidating the Surface Functionality of Biomimetic RGD Peptides Immobilized on Nano-P(3HB-*co*-4HB) for H9c2 Myoblast Cell Proliferation

**DOI:** 10.3389/fbioe.2020.567693

**Published:** 2020-10-27

**Authors:** Sevakumaran Vigneswari, Jun Meng Chai, Khadijah Hilmun Kamarudin, Al-Ashraf Abdullah Amirul, Maria Letizia Focarete, Seeram Ramakrishna

**Affiliations:** ^1^Faculty of Science and Marine Environment, Universiti Malaysia Terengganu, Kuala Terengganu, Malaysia; ^2^School of Biological Sciences, Universiti Sains Malaysia, George Town, Malaysia; ^3^Centre for Chemical Biology, Universiti Sains Malaysia, Bayan Lepas, Malaysia; ^4^Department of Chemistry “Giacomo Ciamician” and INSTM UdR of Bologna, University of Bologna, Bologna, Italy; ^5^Health Sciences and Technologies-Interdepartmental Center for Industrial Research (HST-ICIR), University of Bologna, Ozzano Emilia, Italy; ^6^Department of Mechanical Engineering, Center for Nanofibers and Nanotechnology, National University of Singapore, Singapore, Singapore

**Keywords:** P(3HB-*co*-4HB) nanofibers, RGD peptides, aminolysis, myoblast cells, electrospinning

## Abstract

Biomaterial scaffolds play crucial role to promote cell proliferation and foster the regeneration of new tissues. The progress in material science has paved the way for the generation of ingenious biomaterials. However, these biomaterials require further optimization to be effectively used in existing clinical treatments. It is crucial to develop biomaterials which mimics structure that can be actively involved in delivering signals to cells for the formation of the regenerated tissue. In this research we nanoengineered a functional scaffold to support the proliferation of myoblast cells. Poly(3-hydroxybutyrate-*co*-4-hydroxybutyrate) [P(3HB-*co*-4HB)] copolymer is chosen as scaffold material owing to its desirable mechanical and physical properties combined with good biocompatibility, thus eliciting appropriate host tissue responses. In this study P(3HB-*co*-4HB) copolymer was biosynthesized using *Cupriavidus malaysiensis* USMAA1020 transformant harboring additional PHA synthase gene, and the viability of a novel P(3HB-*co*-4HB) electrospun nanofiber scaffold, surface functionalized with RGD peptides, was explored. In order to immobilize RGD peptides molecules onto the P(3HB-*co*-4HB) nanofibers surface, an aminolysis reaction was performed. The nanoengineered scaffolds were characterized using SEM, organic elemental analysis (CHN analysis), FTIR, surface wettability and their *in vitro* degradation behavior was evaluated. The cell culture study using H9c2 myoblast cells was conducted to assess the *in vitro* cellular response of the engineered scaffold. Our results demonstrated that nano-P(3HB-*co*-4HB)-RGD scaffold possessed an average fiber diameter distribution between 200 and 300 nm, closely biomimicking, from a morphological point of view, the structural ECM components, thus acting as potential ECM analogs. This study indicates that the surface conjugation of biomimetic RGD peptide to the nano-P(3HB-*co*-4HB) fibers increased the surface wettability (15 ± 2°) and enhanced H9c2 myoblast cells attachment and proliferation. In summary, the study reveals that nano-P(3HB-*co*-4HB)-RGD scaffold can be considered a promising candidate to be further explored as cardiac construct for building cardiac construct.

## Introduction

Cardiovascular diseases are the leading cause of death all over the world. Myocardial infarction is commonly caused by the blockage of the coronary artery that prevents the blood flow. This eventually leads to a vast loss of cardiomyocytes (Boffito et al., 2013; Asadpour et al., 2018). The heart has a limited regeneration capacity (Cui et al., 2018) since the myocardial cells usually fail to regenerate tissues after myocardial infarction (Niu et al., 2013). Heart transplantation and use of mechanical assist devices are the best current therapeutic strategy for myocardial infarction; however, these are restricted by the lack of donor organs and complications (Boffito et al., 2013). Thus, significant effort is being devoted in developing alternative therapeutic approaches for developing cardiac construct for building cardiac tissues (Kai et al., 2013; Ravichandran et al., 2013). In this regards, cardiac tissue engineering was introduced as a prospective method to repair and regenerate the infarcted cardiac tissues by developing functional acellular scaffold *in vitro*.

The selection of a suitable biomaterial is crucial in developing functional scaffolds which provide the surface architecture and mechanical support for the proliferation of the cells. Polyhydroxyalkanoates (PHA) are a family of microbial polyesters well-known for their good biocompatibility and tailored absorption rate, making them desirable biomaterial for tissue engineering (Możejko-Ciesielska and Kiewisz, 2016). Among PHAs, poly(3-hydroxybutyrate-*co*-4-hydroxybutyrate) [P(3HB-*co*-4HB)] is the most preferred biomaterial as it’s known to possess supraphysiologic mechanical strength and physical properties as well as minimal foreign body reaction *in vivo* (Sudesh et al., 2000). The control of structural parameters, such as comonomer unit composition and compositional distribution, is known to have an effect on the morphology and physical properties of P(3HB-*co*-4HB), that can be tailored in order to have copolymers that can biodegrade *in vivo* in a predetermined time and manner (Williams et al., 2013). These findings are significant in developing P(3HB-*co*-4HB) as cardiac construct for building cardiac tissues. However, the surface of P(3HB-*co*-4HB) copolymer is hydrophobic. Thus, surface modification to enhance the surface architecture and surface chemistry is required to make this scaffold desirable biomaterial.

The surface architecture of scaffolds depends on the type of biomaterial and the technique employed to fabricate the scaffolds, and it immensely affects the cell-biomaterial interaction. Electrospinning is a simple but effective technology to fabricate nanofibers mimicking the architecture of native extracellular matrix (ECM) which promote cellular behavior and enhance the tissue regeneration (Jin et al., 2012; Awad et al., 2018). Immobilization of biomolecules is a commonly used procedure to add recognition sites on the surface of scaffolds to promote cell adhesion (Woeppel et al., 2018). Aminolysis is a simple chemical modification to introduce amine groups or other functional groups onto the targeted surface via series of chemical reactions (Zhu et al., 2013). Previous studies have shown that biomacromolecule immobilization via aminolysis onto the P(3HB-*co*-4HB) films promoted cell growth and enhanced proliferation (Vigneswari et al., 2016b).

Biomimetic RGD peptides are tri-amino acids (arginine-glycine-aspartic acid) that play crucial role in regulatory functions of many biological activities. Biomimetic RGD peptides are the components of ECM proteins including fibrinogen, collagen, vitronectin and fibronectin which aid in the cell adhesion and specific binding to the transmembrane proteins (Colombo and Bianchi, 2010). Since biomimetic RGD peptides can stimulate cell activity (Kammerer et al., 2011), their incorporation onto appropriate biomaterial surface produces scaffolds that provide significant effects in terms of cell responses. Studies have shown that PHA scaffolds fused with biomimetic RGD peptides enhanced cell adhesion and improved biocompatibility (Dwivedi et al., 2020).

**FIGURE 1:**
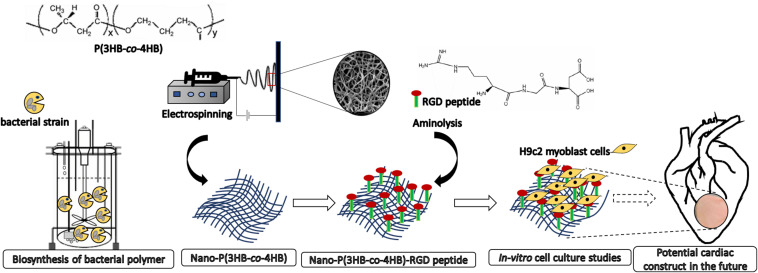
Scheme showing the biosynthesis of bacterial polymer, development of electrospun nano-P(3HB-*co*-4HB) scaffold, immobilization of biomimetic RGD peptides post-electrospinning fabrication and as potential future application for building cardiac tissue.

There is large amount of literature on the surface modification of P(3HB-*co*-4HB) as cellular scaffolds, however, few studies were devoted to the post-electrospinning modification techniques in the context of improving the efficiency of the biomaterials as acellular scaffold. Our study also aims at fabricating acellular scaffold which has the advantages over cellular scaffolds in terms of the off-the-shelf availability for immediate implantation and limited immune reaction (Domenech et al., 2016). In this study, the P(3HB-*co*-4HB) copolymer was biosynthesized using *Cupriavidus malaysiensis* USMAA1020 transformant harboring additional PHA synthase gene. The nano-P(3HB-*co*-4HB) scaffold was fabricated using electrospinning technique and post-electrospinning modification was carried out by conjugating RGD peptides biomolecules onto the surface of electrospun scaffolds via aminolysis (Figure 1). The present study was to determine whether the nano-P(3HB-*co*-4HB)-RGD scaffold would accommodate viability, growth and proliferation of H9c2 myoblast cells to be further developed as potential cardiac construct in the future.

## Materials and Methods

### Materials

RGD peptides (Purity 98.68%) were purchased from GL Biochem Ltd. (Shanghai, China). Ninhydrin assay and 1,6-hexanediamine were purchased from Sigma-Aldrich (United States). Acetic acid and 2-propanol were purchased from JT Baker (United States). Glutaraldehyde was purchased from R&M (Malaysia). H9c2 myoblast cell lines [H9c2(2-1)] (ATCC^®^ CRL-1446^TM^), Dulbecco’s Modified Eagle’s Medium (DMEM) (ATCC^®^ 30-2002^TM^), fetal bovine serum (FBS) (ATCC^®^ 30-2021^TM^) and trypsin/EDTA (ATCC^®^ 30-2101^TM^) were purchased from ATCC. CellTiter 96^®^ AQ_ueous_ One Solution Reagent was purchased from Promega (United States). DMSO was purchased from Invitrogen (United States). Phosphate buffer solution (PBS) and trypan blue stain were purchased from Gibco (United States).

### Biosynthesis of P(3HB-*co*-4HB)

The bacteria strain of *Cupriavidus malaysiensis* USMAA1020 transformant harboring additional PHA synthase gene from *Cupriavidus malaysiensis* USMAA2-4, was used to produce P(3HB-*co*-4HB) copolymer in a 15 L bioreactor with a total working volume of 10 L as previously described (Syafiq et al., 2017). A mixture of carbon precursors (0.75 wt% C) with both 1,4-butanediol (0.625 wt% C) and 1,6-hexanediol (0.125 wt% C) were used for the biosynthesis of P(3HB-*co*-4HB). The biosynthesis was carried out until the measured dry cell weight achieved the highest constant value during the process which was at 84 h. The P(3HB-*co*-4HB) polymer extraction was carried out as previously described (Amirul et al., 2008). Removal of endotoxins were done using hydrogen peroxide (Vigneswari et al., 2015). The gas chromatography (GC) analysis was carried out to determine the PHA composition and content in the lyophilized cells based on a study done by Braunegg et al. (1978) with some modification, using Shimadzu GC-17A (Shimadzu, Japan) as described by Syafiq et al. (2017).

### Fabrication of P(3HB-*co*-4HB) Nanofibers

The electrospinning process was carried out using a custom-built Nano Fiber Production System (NEU-202) instrument as previously described (Vigneswari et al., 2016b). Briefly, the polymer solution was prepared by solubilizing the polymer at a concentration of 8 wt%, in a mixture of dimethylformamide (DMF) and chloroform (ratio 1:4 v/v) which was stirred for 8 h at room temperature. The polymer solution was loaded in 5 mL syringes with metal blunt needle of 21 gauge (G) and diameter of 10 mm. The electrospun nanofibers were collected on a collecting plate positioned at a working distance of 10 cm, perpendicular to the needle tip, and the deposition was performed by automatically sequencing the X-axis of the collector at a speed of 10 mm/s starting from 140 to 165 mm while Y-axis was set at 195 mm. The polymer solution was extruded using a computer-controlled syringe pump, with a flow rate of 1.5 mL/h and an electric potential of 25 kV. The temperature was regulated at 25 ± 2°C with relative humidity of 28 ± 2%.

### Immobilization of Biomimetic RGD Peptide

Immobilization of the RGD peptides onto the nano-P(3HB-*co*-4HB) scaffolds was carried out according to previous work (Vigneswari et al., 2016a). Prior to immobilization, nano-P(3HB-*co*-4HB) scaffolds were thoroughly rinsed with deionized water. Scaffolds were then immersed in 10 wt% 1,6-hexanediamine/2-propanol solution at 37°C and allowed to react. The reaction of 1,6-hexanediamine/2-propanol solution with the scaffolds was carried out for 10, 20, 30, 40, 50, and 60 min. Then, the aminolyzed nano-P(3HB-*co*-4HB) scaffolds were immersed in 1 wt% glutaraldehyde solution at room temperature for 3 h, followed by rinsing with large amount of deionized water. The scaffolds were incubated in RGD solution 2% w/v RGD peptides in 8% v/v acetic acid) at pH 3.4 and at a temperature 2–4*°*C for 24 h. Later, the nano-P(3HB-*co*-4HB)-RGD scaffolds were rinsed with 1% v/v acetic acid solution, followed by three washes in deionized water and dried under vacuum before use. Ninhydrin assay was used to detect the NH_2_ groups present on the aminolyzed nano-P(3HB-*co*-4HB) and nano-P(3HB-*co*-4HB)-RGD scaffolds as previously described (Vigneswari et al., 2015).

### Uptake Efficiency of Biomimetic RGD Peptides

The aminolyzed nano-P(3HB-*co*-4HB) scaffolds were incubated in 3 mL of RGD solution at various concentrations (0.5, 1.0, 1.5, 2.0, and 2.5 mg/mL) at 2–4°C for 24 h. The concentration of RGD immobilized onto the fabricated scaffold (C_r_) was determined by ninhydrin assay. A standard calibration curve was established based on the concentration of RGD solution prior to this. The concentration of RGD in the initial solution (C_i_) was determined based on the calibration curve. The upload efficiency of RGD peptides on the scaffolds was determined by the following equation:

$$\mathrm{Uptake}\mathrm{efficiency}\mathrm{of}\mathrm{RGD}\mathrm{peptides}=\frac{C_{r}}{C_{i}}\times 100\%$$

### Characterization of Nano- P(3HB-*co*-4HB)-RGD Scaffolds

The surface morphology of the scaffolds was observed by scanning electron microscopy (SEM) using FEI Quanta FEG 650. SEM images were used to analyze the fiber diameter by means of the Image Analyzer Olympus CellSens Standard Software. The diameter values of 100 nanofibers, taken in different positions, were measured to obtain the fiber diameter distribution.

The organic elemental analysis (CHN analysis) was carried out to determine the carbon (C), hydrogen (H) and nitrogen (N) content in the scaffolds using the CHNS-O Elemental Analyzer (Thermo Fisher Scientific, United States) as previously described (Vigneswari et al., 2015).

The Fourier transform infrared spectroscopy analysis (FTIR) was recorded with Perkin Elmer Spectrum GX spectrometer. The spectra of each sample were obtained in the range of 4,000–500 cm^–1^ at a resolution of 4 cm^–1^. The spectral outputs were recorded in transmittance as a function of wave number (Salvatore et al., 2018).

The surface wettability of the scaffolds was evaluated using KSV CAM 101 Series Drop Shape Analysis Contact Angle Meter (KSV Instruments Limited, United States). The measurements were repeated three times in different parts of the same scaffold (Salvatore et al., 2018).

Atomic force microscopy (AFM) was conducted using Dimension Edge AFM (Bruker, United States). The resonance frequency was set at 300 kHz. The surface mapping of the scaffolds was standardized at 5 μm × 5 μm (Basnett et al., 2013).

### *In vitro* Degradation of Scaffolds

The initial dry scaffolds were weighed (W_o_) and sterilized under UV for 1 h on each side. The scaffolds were immersed in PBS (pH 7.4) and then incubated under standard cell culture conditions in 5% CO_2_ incubator with 95% relative humidity at 37°C. The PBS media was changed once a week. After the incubation period of 1, 3, 7, 14, 21, 28, and 35 days, the scaffolds were removed from PBS and rinsed with a deionized water. The scaffolds were vacuum dried for 48 h to achieve constant weight. At each incubation time point, the scaffolds were weighed (W_i_) and the percentage of weight loss (% W) at each incubation time was calculated based on the equation below (Ajalloueian et al., 2014).

$$\mathrm{Percentage}\mathrm{of}\mathrm{weight}\left(\%W\right)=100\%-\frac{W_{o}-W_{i}}{W_{o}}\text{×}100\%$$

### Cell Culture

The H9c2 myoblast cell attachment and proliferation studies were carried out to assess the *in vitro* cytotoxicity. The UV sterilized scaffolds were seeded with 1 × 10^5^ cells/mL in 96-well cell culture plate and incubated in a 5% CO_2_ incubator at 37°C for 4 h. The culture of H9c2 myoblast cells on the tissue culture polystyrene plates (T) was used as surface (negative) control. As for *in vitro* cell proliferation evaluation, the cells were seeded at 1 × 10^4^ cells/mL and then incubated in the CO_2_ incubator at 37°C for 24 and 96 h. The cells were washed twice with phosphate buffer solution (PBS). Later, the cells viability for attachment and proliferation were assayed with CellTiter 96^®^ AQ_ueous_ One Solution Reagent containing MTS [3-(4,5-dimethylthiazol-2-yl)-5-(3-carboxymethoxyphenyl)-2-(4-sulfophenyl)-2H-tetrazolium]/PES (phenazine ethosulfate). The absorbance was measured at 490 nm using microplate reader.

### Statistical Analysis

All the data were expressed as mean ± standard deviation (s.d.). The data were analyzed using ANOVA and Tukey’s HSD test using SPSS 20 software. The significance level to consider result significant was set at *p* < 0.05.

## Results and Discussion

### Biosynthesis of P(3HB-*co*-4HB) via Batch Cultivation

The P(3HB-*co*-4HB) was biosynthesized using transformant strain of *Cupriavidus malaysiensis* USMAA1020. The P(3HB-*co*-4HB) content and composition at different time intervals from 24 to 84 h are summarized in Table 1. The results indicate that the highest 4HB molar fraction of 68.0 ± 1.0 mol% was obtained at 84 h of incubation. P(3HB-*co*-4HB) yield increased to 8.0 ± 0.1 g/L at the end of cultivation. The obtained P(3HB-*co*-4HB) content was in the range of 45–69 mol%. As can be seen, the dry cell weight, P(3HB-*co*-4HB) content and PHA yield were considered high due to the synergistic effect of the mixed 4HB precursors of 1,4-butanediol and 1,6-hexanediol cultivation as previously reported by Huong et al. (2017) and Norhafini et al. (2017). It is pointed out that P(3HB-*co*-4HB) with highest 4HB monomer composition is preferred due to the accelerated biodegradability and cellular compatibility induced by the high 4HB molar fraction (Ying et al., 2008; Aziz et al., 2017).

**TABLE 1:** Production of copolymer P(3HB-*co*-4HB) by *Cupriavidus malaysiensis* USMAA1020 transformant^a^.

Time (h)	Dry cell weight (g/L)	PHA content (wt%)^b^	PHA composition (mol%)^b^		PHA yield (g/L)^c^	Residual biomass (g/L)^d^
			3HB	4HB		
24	3.2 ± 0.04^e^	45.4 ± 0.4^e^	84.2 ± 0.3^e^	15.8 ± 0.3^e^	1.4 ± 0.03^e^	1.7 ± 0.02^e^
48	8.2 ± 0.3^f^	57 ± 1.0^f^	61.8 ± 0.5^f^	38.2 ± 0.5^f^	4.6 ± 0.1^f^	3.6 ± 0.2^f^
72	11.5 ± 0.1^g^	64 ± 2.0^g^	34.1 ± 0.05^g^	65.8 ± 0.05^g^	7.4 ± 0.2^g^	4.1 ± 0.3^g^
84	11.6 ± 0.1^g^	69.4 ± 0.3^h^	32 ± 1.0^h^	68 ± 1.0^h^	8.0 ± 0.1^h^	3.5 ± 0.1^f^

*^a^Cell pellets were harvested after 84 h of incubation in a 15-L bioreactor at 200 rpm, 1 vvm at 30°C with an initial pH 7.0. ^b^Determined from GC analysis. ^c^Calculated based on dry cell weight x PHA content. ^d^Calculated by subtracting PHA yield from dry cell weight. ^e–h^Different superscript within the same column are significantly different at p < 0.05 level (Tukey’s HSD test). Data show the mean ± standard deviation of triplicates (n = 3).*

### Fabrication of Nano-P(3HB-co-4HB) and Immobilization of Biomimetic RGD Peptides

**FIGURE 2:**
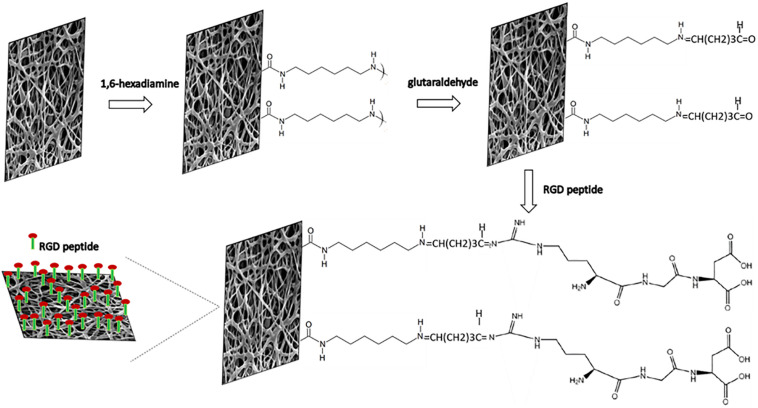
Schematic diagram of the immobilization reactions of RGD peptides onto nano-P(3HB-*co*-4HB) scaffold via aminolysis.

Electrospinning is a versatile method to fabricate nanofibrous scaffolds which closely mimic the ECM architecture (Lakshmanan et al., 2012; Jun et al., 2018). The smooth, uniform and beadless P(3HB-*co*-4HB) nanofibers were fabricated after a careful optimization of the polymer solution concentration and processing parameters (applied voltage and flow rate). The final conditions were polymer concentration 8 wt%, applied voltage 25 kV and flow rate 1.5 mL/h, in agreement with those reported by Chai et al. (2020). However, as already anticipated, these scaffolds lack biological cell recognition sites which restricts the cell-material interaction limiting their applications. One of the effective surface modification strategies is the bioconjugation of biomolecules onto the scaffold surface which enhances biorecognition and induces cell attachment as well as cell maturation (Pettikiriarachchi et al., 2012). The step-by-step immobilization of RGD peptides on nano-P(3HB-*co*-4HB) scaffold via aminolysis is schematically illustrated in Figure 2. The aminolysis of nano-P(3HB-*co*-4HB) was conducted using 1,6-hexanediamine in 2-propanol to introduce amino functionalities which serve as the linker to immobilize the biomimetic RGD peptide onto the scaffold surface. The 1,6-hexanediamine molecules enable one amino group to attack the ester bond of the nano-P(3HB-*co*-4HB) scaffold while the other serves as a potential anchoring site of biomolecules.

In the mechanism of aminolysis reaction, the cleavage of ester bonds takes place, and one amino group of 1,6-hexanediamine reacts with one carbonyl group of nano-P(3HB-*co*-4HB) to form a covalent amide bond (Zhu et al., 2013). Prior to the immobilization of RGD peptides, the aminolyzed-nano-P(3HB-*co*-4HB) scaffold was treated with glutaraldehyde. A bond of –N=CH–(CH_2_)_3_CHO is formed between the NH_2_ group of the aminolyzed scaffold and the aldehyde group of glutaraldehyde. Basically, glutaraldehyde is used as a coupling agent to covalently link the RGD peptides at the aminolyzed-nano-P(3HB-*co*-4HB), thus immobilizing the RGD peptides onto the scaffold surface (Vigneswari et al., 2016a).

The RGD peptides content and uptake efficiency of the immobilized RGD peptides onto the scaffold surface are shown in Table 2. It was observed that the amount of RGD immobilized on the scaffold increased as the concentration of RGD increased, and the highest RGD content (0.6 mg/cm^2^) was observed when the concentration of RGD was 2.0 mg/mL. The same behavior was found for the uptake efficiency of RGD immobilized, that increased up to 82.8 ± 1.4% with the increase of RGD concentration from 0.5 to 2.0 mg/mL. It is worth noting that there was a decrease in RGD content, as well as in the uptake efficiency of RGD immobilized, beyond the RGD concentration of 2.0 mg/mL. This result might be attributed to the limited number of binding sites onto the scaffold surface. Hence, 2.0 mg/mL was the optimum RGD concentration used in this study with the highest uptake efficiency of RGD immobilized onto the nano-P(3HB-*co*-4HB) scaffold. This result indicates that RGD peptides can be immobilized onto surface of biomaterials in different amount (Zheng et al., 2014; Li et al., 2017).

**TABLE 2:** The uptake efficiency of RGD immobilized onto the scaffolds determined by ninhydrin test.

Scaffold^a^	RGD content (mg/cm^2^)^c^	Uptake efficiency of RGD immobilized on the scaffold (%)
Nano-P(3HB-*co*-4HB)^b^	0^d^	0^d^
Nano-P(3HB-*co*-4HB)/0.5 mg/mL RGD	0.20 ± 0.01^e^	54.0 ± 2.5^e^
Nano-P(3HB-*co*-4HB)/1.0 mg/mL RGD	0.20 ± 0.03^e^	61.1 ± 2.8^f^
Nano-P(3HB-*co*-4HB)/1.5 mg/mL RGD	0.30 ± 0.02^f^	69.5 ± 0.9^g^
Nano-P(3HB-*co*-4HB)/2.0 mg/mL RGD	0.60 ± 0.03^g^	82.8 ± 1.4^h^
Nano-P(3HB-*co*-4HB)/2.5 mg/mL RGD	0.50 ± 0.04^h^	78.4 ± 0.9^h^

*^a^Surface area of the fabricated circular scaffold is 12.6 cm^2^. ^b^P(3HB-co-68 mol% 4HB) was used as a control. ^c^RGD content = amount of RGD in 1 cm^2^ × 12.6 cm^2^. ^d–h^ Different superscript within the same column are significantly different at p < 0.05 level (Tukey’s HSD test). Data show the mean ± standard deviation of triplicates (n = 3).*

### Characterization of Nano- P(3HB-*co*-4HB)-RGD Scaffolds

**FIGURE 3:**
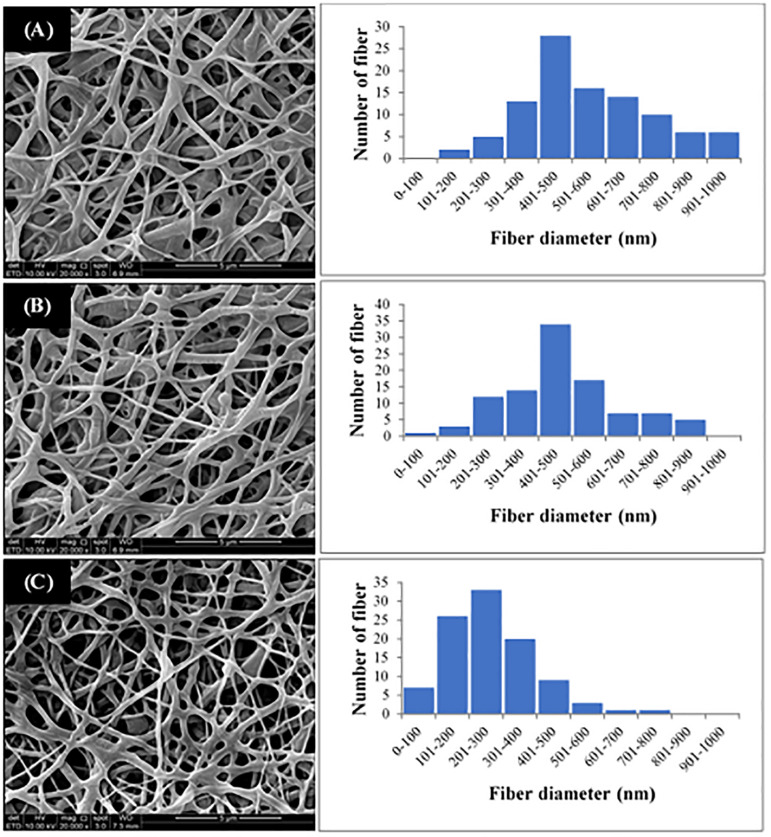
SEM images and fiber diameter distribution of **(A)** Nano-P(3HB-*co*-4HB), **(B)** NH_2_-nano-P(3HB-*co*-4HB), and **(C)** Nano-P(3HB-*co*-4HB)-RGD scaffolds. The magnification of SEM images is 20 kX.

The P(3HB-*co*-4HB) copolymer was successfully electrospun into scaffold with smooth and beadless nanofibers, as shown in Figure 3. The fiber diameter of the fabricated scaffolds was measured using image analysis software and the fiber diameter distribution was obtained (Figure 3). Results showed that the average fiber diameter of the nano-P(3HB-*co*-4HB)-RGD scaffold was in the range 201–300 nm, whereas both nano-P(3HB-*co*-4HB) and NH_2_-nano-P(3HB-*co*-4HB) scaffolds showed an higher fiber average diameter, in the range 401–500 nm. Therefore, the addition of RGD peptides onto the scaffold decreased the diameter of the nanofibers. This result might be explained assuming that, due to the intrinsic mechanism of aminolysis reaction, during the whole functionalization process erosion of the polyester surface inevitably takes place, thus reducing fiber diameter (Zhu et al., 2013).

It is worth noting that the average fiber diameter of the fabricated scaffolds was comparable to that of ECM fibers, that has been reported to be in the range of 50–500 nm (Barnes et al., 2007), therefore the nano-P(3HB-*co*-4HB)-RGD scaffolds are morphologically biomimetic of ECM and can be considered as ideal ECM analogs.

As for the CHN analysis, it is used to determine the percentages of carbon, hydrogen and nitrogen elements present on the scaffolds. Alternatively, the CHN analysis can be used to determine if the RGD peptides were immobilized onto the scaffold. Based on results reported in Table 3, the nano-P(3HB-*co*-4HB) scaffold contains both carbon and hydrogen but there was no nitrogen element present as P(3HB-*co*-4HB) copolymer does not naturally contain nitrogen element. The aminolyzed nano-P(3HB-*co*-4HB) and nano-P(3HB-*co*-4HB)-RGD, on the other hand, show the presence of nitrogen due to the functional amine groups and RGD peptide respectively. Similarly, study by Kim et al. (2014) and Bhattacharjee et al. (2015) proved that aminolyzed polymer scaffold were obtained based on the presence of nitrogen atoms.

**TABLE 3:** CHN analysis of the scaffolds.

Scaffold	Carbon (%)	Hydrogen (%)	Nitrogen (%)
Nano-P(3HB-*co*-4HB)	51.3 ± 1.3^a^	7.4 ± 1.1^a^	0^a^
NH_2_-nano-P(3HB-*co*-4HB)	47.7 ± 2.4^ab^	6.6 ± 0.6^a^	15.7 ± 2.7^b^
Nano-P(3HB-*co*-4HB)-RGD	46.4 ± 1.5^b^	6.1 ± 0.2^a^	17.6 ± 0.6^b^

*P(3HB-co-68 mol% 4HB) was used. ^a,b^Different superscript within the same column are significantly different at p < 0.05 level (Tukey’s HSD test). Data shows the mean ± standard deviation of triplicates (n = 3).*

**FIGURE 4:**
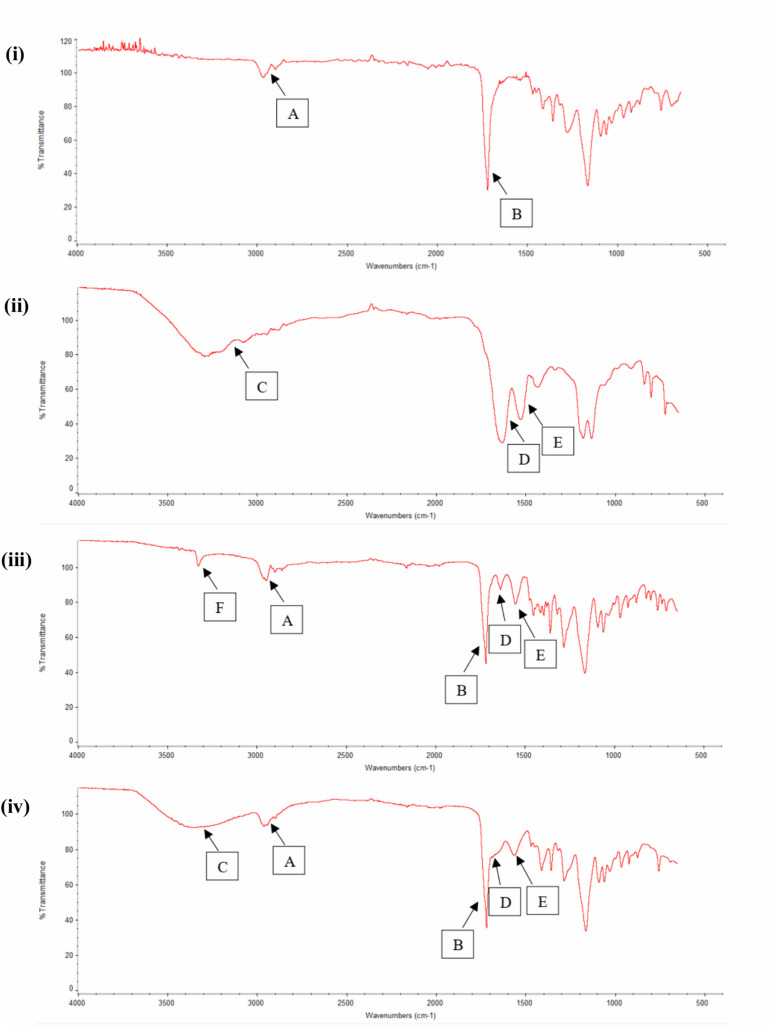
FTIR spectra of (i) Nano-P(3HB-*co*-4HB), (ii) RGD peptides, (iii) NH_2_-nano-P(3HB-*co*-4HB), and (iv) Nano-P(3HB-*co*-4HB)-RGD. In the FTIR spectra, A indicates CH_3_ and CH_2_ stretching, B indicates C=O stretching, C indicates N–H stretching (broad band), D indicates amide I, E indicates amide II and F indicates N–H stretching (small and strong intensity band).

FTIR study was also used as an evidential analysis for the immobilization of RGD peptides on the nano-P(3HB-*co*-4HB) scaffold as shown in Figure 4. The FTIR spectrum of nano-P(3HB-*co*-4HB) scaffold showed two transmittance bands at 2,965 cm^–1^ and 2,898 cm^–1^ which attributed to stretching vibration of C–H bonds of methyl group (CH_3_) and methylene group (CH_2_) respectively. It also exhibited the intense absorption band at 1,717 cm^–1^, corresponding to the ester carbonyl group (C=O), which is the main functional group of P(3HB-*co*-4HB) (Vigneswari et al., 2016a). In the FTIR spectrum of RGD peptides, a broad band assigned to the N–H stretching was present at 3,292 cm^–1^, while the characteristic absorption bands at 1,633 and 1,526 cm^–1^ correspond to the amide I and amide II respectively. Accordingly, the FTIR spectrum of nano-P(3HB-*co*-4HB)-RGD scaffold, shows the relevant bands attributed to the nano-P(3HB-*co*-4HB) and to RGD peptides, indicating that that the RGD peptides were successfully immobilized on the nano-P(3HB-*co*-4HB) scaffold.

The water contact angle of the scaffolds was found to decrease in the order of nano-P(3HB-*co*-4HB) > NH_2_-nano-P(3HB-*co*-4HB) > nano-P(3HB-*co*-4HB)-RGD (Table 4). In general, large contact angles (>90°) correspond to hydrophobic behavior with low wettability whereas small contact angles (<90°) correspond to high wettability (Kurusu and Demarquette, 2019; Jeznach et al., 2019). The obtained results indicate that, as expected, the hydrophilicity increased as the NH_2_ groups were introduced onto the scaffold and the incorporation of RGD peptides further enhanced the wettability of the scaffold. The nano-P(3HB-*co*-4HB)-RGD scaffold exhibited the lowest water contact angle of 14.7 ± 1.5°. This is attributed to the presence, in the RGD peptides, of hydrophilic groups which includes –NH_2_ and –COOH. Similar trend has been reported by Guler et al. (2016).

**TABLE 4:** Surface wettability of the fabricated scaffolds.

Scaffold	Water contact angle (°)	Image
Nano-P(3HB-*co*-4HB)	39.3 ± 1.9	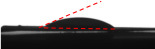
NH_2_-nano-P(3HB-*co*-4HB)	28.3 ± 2.1	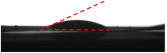
Nano-P(3HB-*co*-4HB)-RGD	14.7 ± 1.5	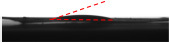

*Data show the mean ± standard deviation of triplicates (n = 3).*

The surface topography and roughness of the scaffolds were analyzed using AFM and results are shown in Table 5. The surface of the nano-P(3HB-*co*-4HB) showed a smoother surface with less protuberances whereas nano-P(3HB-*co*-4HB)-RGD scaffold has the roughest surface, containing more protuberances as compared to the other scaffolds. However, the nano-P(3HB-*co*-4HB)-RGD and NH_2_-nano-P(3HB-*co*-4HB) exhibited relatively similar average surface roughness of 0.06 ± 0.02 μm and 0.05 ± 0.004 μm respectively, as compared to nano-P(3HB-*co*-4HB) scaffold. The increase of roughness in the functionalized fiber surface is possibly attributed to the chemical reaction used to introduce both amine groups and RGD peptides onto the surface (Kim et al., 2014; Bhattacharjee et al., 2015; Kaerkitcha et al., 2016).

**TABLE 5:** Surface roughness and topography of the fabricated scaffolds.

Scaffold	Surface roughness^a^		Image
	Root mean square roughness, R_q_ (μm)	Average roughness, R_a_ (μm)	
Nano-P(3HB-*co*-4HB)	0.03 ± 0.02^b^	0.02 ± 0.01^b^	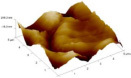
NH_2_-nano-P(3HB-*co*-4HB)	0.07 ± 0.01^bc^	0.05 ± 0.004^bc^	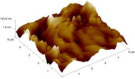
Nano-P(3HB-*co*-4HB)-RGD	0.07 ± 0.02^c^	0.06 ± 0.02^c^	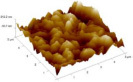

*^a^Calculated from AFM based on the standard formula integrated in the software. ^b,c^Different superscript within the same column are significantly different at p < 0.05 level (Tukey’s HSD test). Data shows the mean ± standard deviation of triplicates (n = 3).*

**FIGURE 5:**
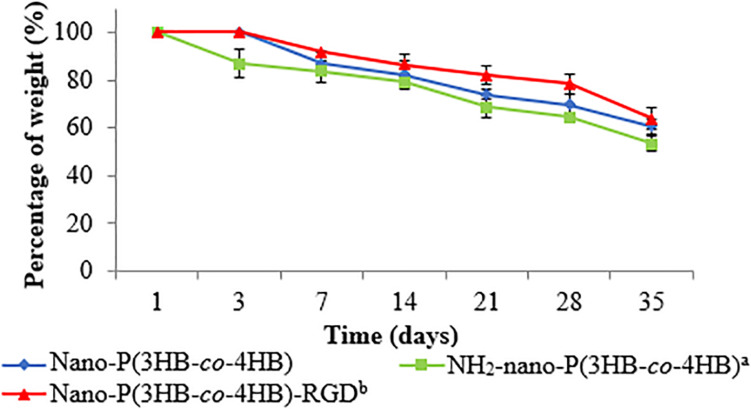
*In vitro* degradation of the fabricated scaffold as a function of time. The data show the mean ± standard deviation of triplicates (*n* = 3).

One of the desirable properties of biomaterial is biodegradability. The *in vitro* degradation study was performed by evaluating the percentage of weight of the scaffold at the end of the degradation as shown in Figure 5. Interestingly, nano-P(3HB-*co*-4HB)-RGD scaffold exhibited significantly lowest degradation as compared to NH_2_-nano-P(3HB-*co*-4HB) scaffolds. The study by Asadpour et al. (2018) indicated that cardiac graft should be biodegraded over the period of regeneration of myocardium after myocardial infarction, which usually takes approximately 6–8 weeks, in order to prevent the formation of fibrous capsule and severe inflammatory reactions. Based on the result obtained, nano-P(3HB-*co*-4HB)-RGD scaffold showed the lowest degradation rate with respect to the other scaffolds tested and experienced a weight loss of about 36% after 35 days. Thus, it can be postulated that nano-P(3HB-*co*-4HB)-RGD scaffold can retain sufficient structural integrity to provide a favorable environment to support cardiac cell growth for potential cardiac tissue engineering.

### Evaluation of *in vitro* Attachment and Proliferation of Myoblast Cells

**FIGURE 6:**
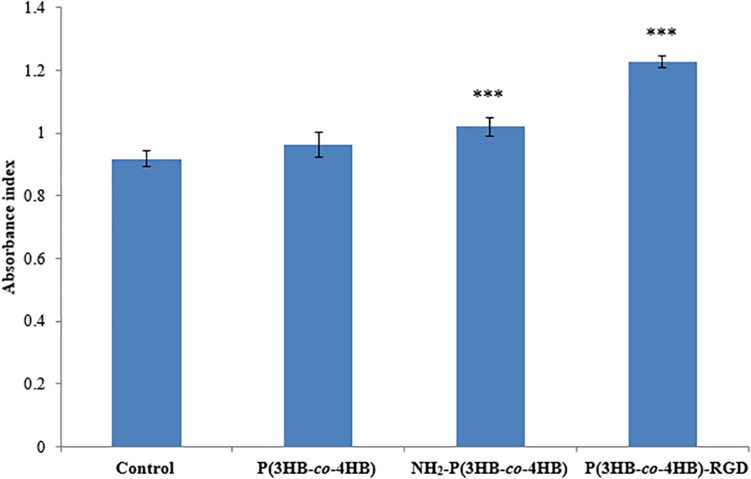
Attachment of H9c2 myoblast cells on the scaffolds at 4 h after cell seeding. TCPS plate was used as control. Data show the mean ± standard deviation of six replicates. Statistically significant difference is indicated with ***p < 0.05 (Tukey’s HSD test) (*n* = 6).

Figure 6 shows the attachment of H9c2 myoblast cells on the scaffolds after 4 h of cell seeding. The nano-P(3HB-*co*-4HB)-RGD scaffold exhibited significantly highest absorbance value of cell attachment. Based on the results obtained it can be deduced that the attachment density of cells seeded on the TCPS, P(3HB-*co*-4HB), NH_2_-P(3HB-*co*-4HB), and nano-P(3HB-*co*-4HB)-RGD. This finding is in line with previous results demonstrating that the addition of RGD peptide enhances cell attachment (Zhang et al., 2009).

**FIGURE 7:**
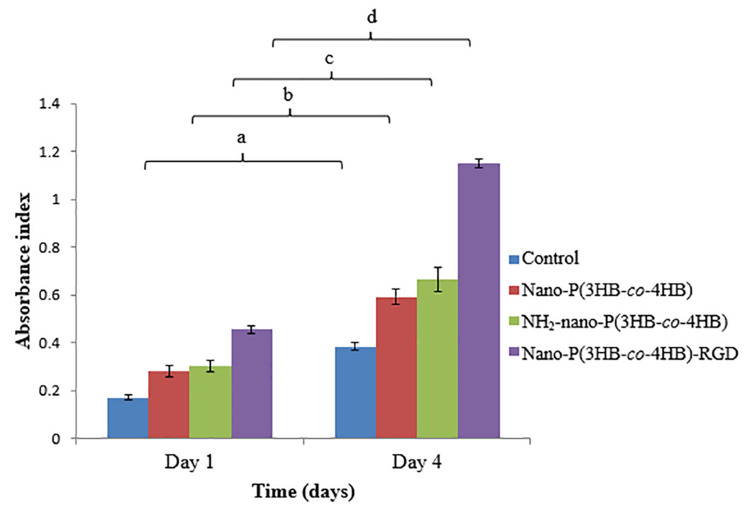
Proliferation of H9c2 myoblast cells on the scaffolds at day 1 and day 4 after cell seeding. TCPS as a control. The data show the mean ± standard deviation of six replicates. Mean data accompanied by different letters indicates significant difference within each respective group (Tukey’s HSD test, *p* < 0.05) (*n* = 6).

The proliferation of H9c2 myoblast cells on the scaffolds for 4-day were shown in Figure 7. The NH_2_-nano-P(3HB-*co*-4HB) scaffold exhibited higher H9c2 myoblast cell proliferation than the nano-P(3HB-*co*-4HB) scaffold. It can be deduced that NH_2_ groups on the scaffold surface improves cell adhesion and proliferation as compared to the scaffold surface before aminolysis reaction (Dallan, 2017).

Meanwhile, the nano-P(3HB-*co*-4HB)-RGD scaffold exhibited 2.0 folds and 1.8 folds increase of H9c2 myoblast cells on day 4 as compared to nano-P(3HB-*co*-4HB) and NH_2_-nano-P(3HB-*co*-4HB) scaffold respectively. It was noted that RGD peptide is an essential component of the scaffolds to improve the cellular attachment and proliferation, particularly for cardiac cells (Shachar et al., 2011).

Considering the obtained results, the enhancement of the surface properties after immobilization of RGD peptides onto the scaffold surface via aminolysis, indicates that RGD peptides is effective in promoting cell attachment and proliferation.

As discussed above, in this work it was found that the attachment and proliferation of H9c2 myoblast cells increased in the order of nano-P(3HB-*co*-4HB) < NH_2_-nano-P(3HB-*co*-4HB) < nano-P(3HB-*co*-4HB)-RGD which is opposite to the fiber diameter trend, which decreased in this order. This indicated that the nano-P(3HB-*co*-4HB)-RGD scaffold which possessed smallest fiber diameter achieved the higher myoblast cell attachment and proliferation. This could be explained considering that the nano-P(3HB-*co*-4HB)-RGD scaffold is characterized by the smallest fiber diameter range of 200–300 nm that closely mimics the ECM scale (Wissing et al., 2017). Moreover, nano-P(3HB-*co*-4HB)-RGD scaffold exhibited the smallest water contact angle value that corresponds to the high hydrophilic surface. Hydrophilic surface directly affects the cell survival, adhesion and proliferation (Ma et al., 2017). The presence of RGD peptides shifted the surface properties of the P(3HB-co-4HB) nanofiber scaffold from hydrophobic to hydrophilic, that gives rises to the higher myoblast cell attachment and proliferation on the RGD-modified scaffold.

The immobilization of RGD peptides not only enhanced the hydrophilic surface, but also increased the cell recognition sites for cell attachment and proliferation. The immobilized RGD peptides provide an adhesive interface between the scaffold and cells that promotes the cell-scaffold interactions (Wang et al., 2013). The conjugation of RGD peptides on the functionalized surface of nano-P(3HB-*co*-4HB) scaffold, could be probably recognized by the cellular integrin as the main binding domain within ECM proteins, thereby stimulating the cell signals that improves the myoblast cell attachment and proliferation on the nano-P(3HB-*co*-4HB)-RGD scaffold.

Though NH_2_-nano-P(3HB-*co*-4HB) and nano-P(3HB-*co*-4HB)-RGD scaffold did not show significant difference in the highest surface roughness but nano-P(3HB-*co*-4HB)-RGD scaffold enhanced attachment and proliferation of myoblast cells. This indicates that conjugation of RGD peptide enhances cells to anchor, grow, proliferate and allows cells to migrate and populate (Thuaksuban et al., 2011; Venkatesan et al., 2014). However, increased cell adhesion and proliferation would not obtain if unsuitable biomaterial is used despite the improvement in the surface properties of this biomaterial. The study by Mu et al. (2015) demonstrated that P(3HB-*co*-4HB) was an excellent biomaterial that supported the engraftment and proliferation of the transplanted cells in the damaged myocardium for long term periods. The synergistic impact between P(3HB-*co*-4HB) nanofibers and RGD peptides enhanced and improved the cell-scaffold interactions that resulted in the higher attachment and proliferation of H9c2 myoblast cells.

**FIGURE 8:**
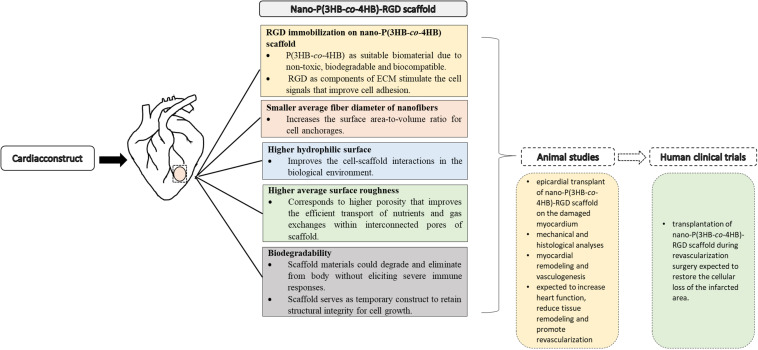
Approaches to cardiac tissue engineering using nano-P(3HB-*co*-4HB)-RGD scaffold as potential acellular scaffold, toward future animal studies and human clinical trials.

The promising results achieved indicate that the nano-P(3HB-*co*-4HB)-RGD peptide is a suitable material that will be envisioned to emerge as potential cardiac construct for building cardiac tissue in the future and facilitate into clinical translation (Figure 8).

## Conclusion

In this study we demonstrated that the fabrication of P(3HB-*co*-4HB) nanofiber scaffold through electrospinning, and the immobilization of RGD peptides onto scaffold surface through aminolysis, were efficient surface modification strategies to improve the biomimentic characteristics of the scaffold. The morphological characterization of the functionalized scaffolds was performed by means of SEM and AFM analysis that demonstrated that the nano-P(3HB-*co*-4HB)-RGD scaffold possessed fibers with the smallest average fiber diameter distribution, in the range of ECM fibers and with higher roughness with respect to non-functionalized fibers. The chemical analysis, carried out through CHN and FTIR, confirmed that RGD peptides were efficiently immobilized and a peptide quantification was performed. Surface wettability and *in vitro* degradation evaluation were conducted to further assess surface hydrophilicity and degradation rate, that are important properties in view of *in vitro* testing. It was shown that RGD functionalization significantly affects the biocompatibility of the scaffold and promoted the cell-scaffold interaction. Nano-P(3HB-*co*-4HB)-RGD scaffold showed the highest attachment and proliferation of H9c2 myoblast cells. Hence, P(3HB-*co*-4HB)-RGD nanofiber scaffold could be an excellent biomaterial to be potentially developed into acellular scaffold for cardiac construct. Further *in vivo* studies are needed to investigate the feasibility and applicability of P(3HB-*co*-4HB)-RGD nanofiber scaffold in the biomedical field.

## Data Availability Statement

The raw data supporting the conclusions of this article will be made available by the authors, without undue reservation, to any qualified researcher.

## Author Contributions

All authors listed have made a substantial, direct and intellectual contribution to the work, and approved it for publication.

## Conflict of Interest

The authors declare that the research was conducted in the absence of any commercial or financial relationships that could be construed as a potential conflict of interest.
